# Supplementary material for: Pilot Study Exploring the Perspectives of Canadian Clients Who Received Digitally Delivered Psychotherapies Utilized for Trauma-Affected Populations
Source: Int J Environ Res Public Health. 2025 Feb 4;22(2):220. doi: 10.3390/ijerph22020220 (PMC11855895; doi:10.3390/ijerph22020220)
Supplement: Supplementary file 1 [file ijerph-22-00220-s001.zip › ijerph-3313395-supplementary-proof/Client Paper Supplementary Files/File S1. Copy of AQMH and UTAUT Models and Surveys, Client Paper.docx]

**Figure S1**. Copy of the Alberta Quality Matrix for Health [25]


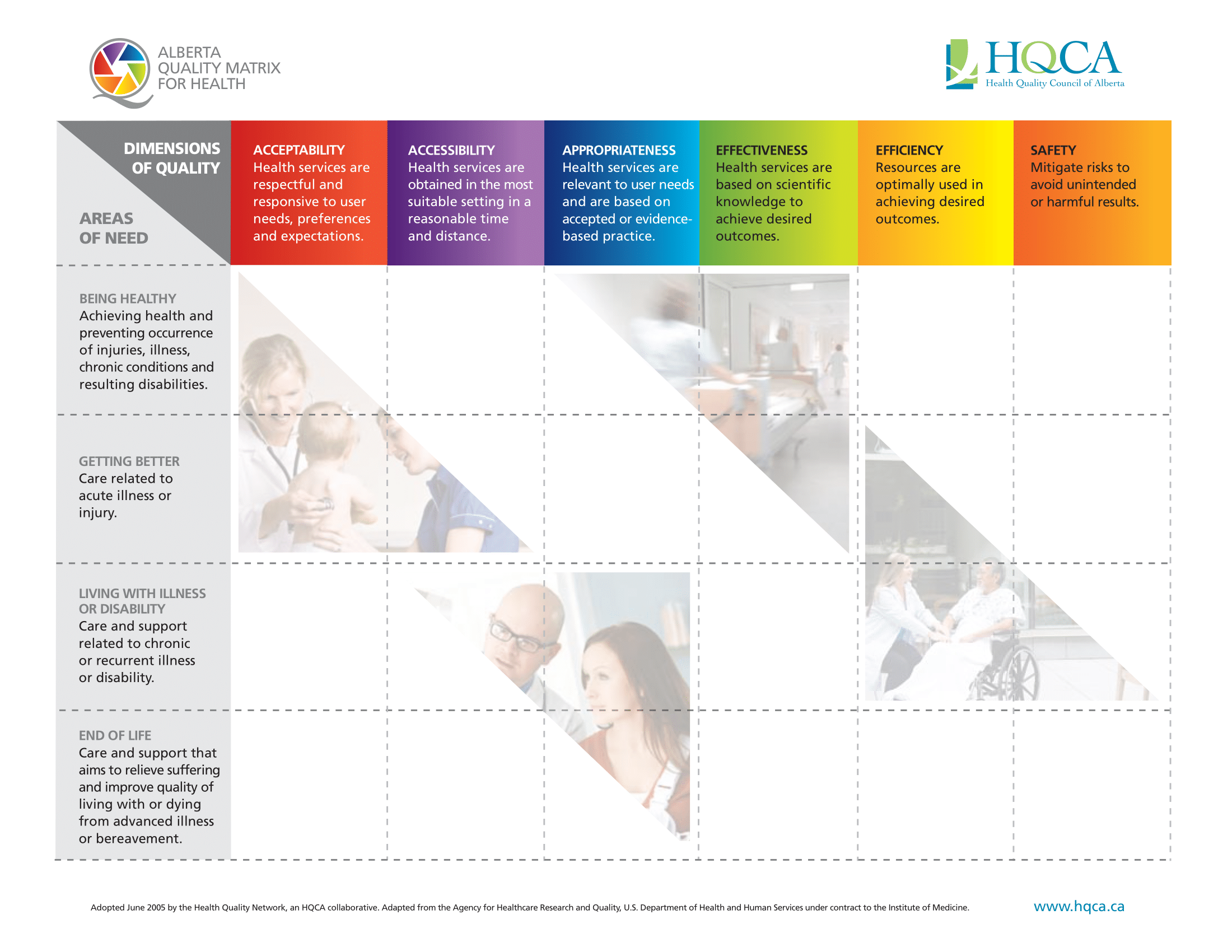


**Quality Matrix for Health Questionnaire - Digitally Delivered Trauma Therapy Client Version**

Instructions

The Quality Matrix for Health Questionnaire Tool is used to evaluate the quality-of-service delivery based on 10 criteria, which appear below. For each criterion, please provide a rating for in person and digital delivery of trauma therapy using a scale of 1 (low) to 7 (high) and offer comments/an explanation ONLY if you wish.

- 1. Ease of use refers to the degree to which it can be used without much effort.

In person: : 1---2---3---4---5---6---7

Digital: 1---2---3---4---5---6---7

Comments/Explanation:

- 1. Convenience refers to the degree to which it saves or simplifies work, and adds to one's ease or comfort.

In person: : 1---2---3---4---5---6---7

Digital: 1---2---3---4---5---6---7

Comments/Explanation:

- 1. Acceptability refers to whether it was respectful and responsive to user needs, preferences and expectations.

In person: : 1---2---3---4---5---6---7

Digital: 1---2---3---4---5---6---7

Comments/Explanation:

- 1. Practicality refers to how feasible it is.

In person: : 1---2---3---4---5---6---7

Digital: 1---2---3---4---5---6---7

Comments/Explanation:

- 1. Accessibility refers to whether it is delivered in a suitable setting in a reasonable time and distance

In person: : 1---2---3---4---5---6---7

Digital: 1---2---3---4---5---6---7

Comments/Explanation:

- 1. Appropriateness refers to whether it is relevant to patient needs.

In person: : 1---2---3---4---5---6---7

Digital: 1---2---3---4---5---6---7

Comments/Explanation:

- 1. Effectiveness refers to whether it helps to achieve desired outcomes.

In person: : 1---2---3---4---5---6---7

Digital: 1---2---3---4---5---6---7

Comments/Explanation:

- 1. Efficiency refers to whether it uses resources optimally to achieve desired outcomes.

In person: : 1---2---3---4---5---6---7

Digital: 1---2---3---4---5---6---7

Comments/Explanation:

- 1. Safety refers to whether it reduces risks and avoids unintended or harmful results.

In person: : 1---2---3---4---5---6---7

Digital: 1---2---3---4---5---6---7

Comments/Explanation:

- 1. Fit refers to how well it aligns with initiatives, structures, and supports within clinics/Alberta Health Services, as well as the priorities of patients and clinicians.

In person: : 1---2---3---4---5---6---7

Digital: 1---2---3---4---5---6---7

Comments/Explanation:

General Paragraph Text Boxes:

In light of the above factors 1-10 regarding the digital delivery of trauma therapy for you, your organization and patients:

What works well and for whom? Please explain.

What doesn’t work well and for whom? Please explain.

What lessons were learned from the rapid transition to digital delivery?

What would you recommend? Please explain.

**Figure S2**. Copy of the Unified Theory of Acceptance and Use of Technology (UTAUT) Model [26]

| **Digital Delivery of Mental Health Therapy – For Clients WITH Experience with Digital Therapy** | | | | | | | |
| --- | --- | --- | --- | --- | --- | --- | --- |
| The purpose of this survey is to gather data on what factors affect client use of a digital system to deliver mental health therapy during the therapy sessions. Please indicate your level of agreement with each item. Mark only one X in a box per item. In the questions below, “digital delivery of therapy” means doing mental health therapy sessions remotely, with the therapist and patient at different locations but talking with each other over the internet or a telephone. | | | | | | | |
| **Item** | **Strongly disagree (1)** | **Disagree (2)** | **Slightly disagree**  **(3)** | **Neither agree nor disagree (4)** | **Slightly agree**  **(5)** | **Agree**  **(6)** | **Strongly agree**  **(7)** |
| 1. Using digital delivery of therapy improved my mental health (PE-PU1) |  |  |  |  |  |  |  |
| 2. Using digital delivery of therapy had a positive effect on my mental health (PE-JF2) |  |  |  |  |  |  |  |
| 3. Using digital delivery of therapy has improved my quality of life (PE-JF3) |  |  |  |  |  |  |  |
| 4. Interacting with the system used for digital delivery of therapy was easy for me (EE-EU1) |  |  |  |  |  |  |  |
| 5. Interacting with a digital system to receive therapy was clear and understandable (EE-EU2) |  |  |  |  |  |  |  |
| 6. The system used for digital delivery of therapy was easy to use for me |  |  |  |  |  |  |  |
| 7. People who are important to me think that I should be involved in using digital delivery of therapy (SI-SN1) |  |  |  |  |  |  |  |
| 8. I used digital delivery of therapy because my colleagues used it too (SI-SF2) |  |  |  |  |  |  |  |
| 9. In general, my organization supported my use of digital delivery of therapy (SI-SF3) |  |  |  |  |  |  |  |
| 10. Guidance was available to me during my interaction with the system used for digital delivery of therapy (FC-FC1) |  |  |  |  |  |  |  |
| 11. Specialized instruction on how to use the system used for digital delivery of therapy was available to me (FC-FC2) |  |  |  |  |  |  |  |
| 12. A specific person (or group of people) was available to assist with any difficulties with the digital delivery of therapy (FC-FC3) |  |  |  |  |  |  |  |
| 13. I am willing to use digital delivery of therapy in the future (BI1) |  |  |  |  |  |  |  |
| 14. I plan to use digital delivery of therapy in the future (BI2) |  |  |  |  |  |  |  |
| 15. I predict I will use digital delivery of therapy in the future (BI3) |  |  |  |  |  |  |  |
| 16. I used digital delivery of therapy to explore an alternative way to improve my quality of life (U1) |  |  |  |  |  |  |  |
| 17. I used digital delivery of therapy to explore an alternative way to improve my mental health (U2) |  |  |  |  |  |  |  |
| 18. I used digital delivery of therapy to explore an alternative way of having a positive effect on my mental health (U3) |  |  |  |  |  |  |  |
