# Supplementary material for: Pilot Study Exploring the Perspectives of Canadian Clients Who Received Digitally Delivered Psychotherapies Utilized for Trauma-Affected Populations
Source: Int J Environ Res Public Health. 2025 Feb 4;22(2):220. doi: 10.3390/ijerph22020220 (PMC11855895; doi:10.3390/ijerph22020220)
Supplement: Supplementary file 1 [file ijerph-22-00220-s001.zip › ijerph-3313395-supplementary-proof/Client Paper Supplementary Files/File S2. Client Interview Script.docx]

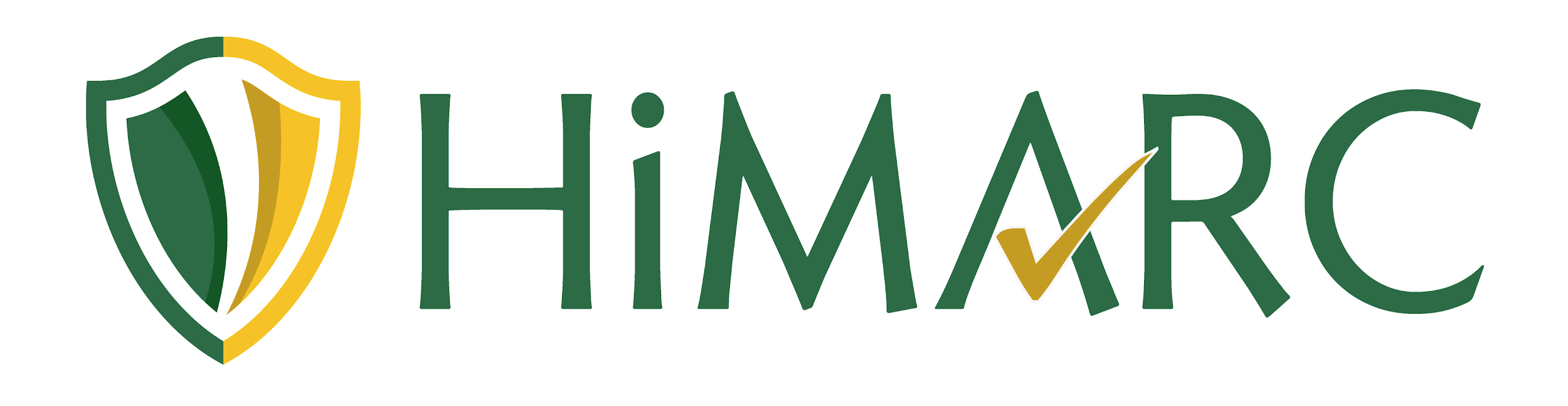


Semi-Structured Interview and Focus Group Questions – **Clients**

COVID-19 Physical Distancing, Virtual Delivery of Trauma Therapies to Trauma-Affected Populations

This interview/focus group follows an iterative process (additional questions may be asked based on the answers to the questions within this guide).

1. Can you tell us about how the shift from in-person trauma therapy to digital delivery occurred for you?
2. Were you given a choice about moving to DH or was this required/forced because of COVID-19?
   - 1. If this change was a choice, why did you choose to move to DH?
     2. If this change was not a choice, how did/do you feel about this?
3. When you moved to DH delivery, did you have any concerns, worries, or hesitations?
   - 1. If yes, were you given opportunities to discuss these concerns?
     2. Who initiated the conversation?
     3. Have you been able to review these concerns throughout your treatment?
4. What has your experience been like receiving trauma therapy via DH?
5. Are there elements of receiving trauma therapy via DH that you feel have changed?
   - 1. Do you think these changes are making receiving trauma treatment better or worse?
6. Do you think there may be certain time periods of treatment (i.e. beginning, middle, end) where DH may be more or less conducive?
7. Do you think there may be certain people or personalities from whom DH may be more or less conducive? Where do you see yourself on this spectrum? What about your therapist?

1. In your experience, are there certain types of trauma therapy that may be more or less conducive to DH?
2. Have you ever had an experience of concerns about your safety, emotional regulation, or follow-up effects from your trauma therapy session?
3. What considerations or advice would you tell someone who was considering receiving trauma therapy via DH?
4. In your opinion/experience, what could be improved/done differently to better serve TAPs in regard to DH delivery of trauma therapy? What are the problems that need to be addressed?
