# Supplementary material for: Pilot Study Exploring the Perspectives of Canadian Clients Who Received Digitally Delivered Psychotherapies Utilized for Trauma-Affected Populations
Source: Int J Environ Res Public Health. 2025 Feb 4;22(2):220. doi: 10.3390/ijerph22020220 (PMC11855895; doi:10.3390/ijerph22020220)
Supplement: Supplementary file 1 [file ijerph-22-00220-s001.zip › ijerph-3313395-supplementary-proof/Client Paper Supplementary Files/File S3. Client Summary of Statistical Tests.docx]

| **Table S1. Client Alberta Quality Matrix for Health median score paired sample Wilcoxon signed-rank test summary comparing median scores for digital delivery vs in-person therapy.** | | | | |
| --- | --- | --- | --- | --- |
|  | Null Hypothesis | Test | Sig.^a,b^ | Decision |
| 1 | The median of E_o_U_T equals .00. | One-Sample Wilcoxon Signed Rank Test | .201 | Retain the null hypothesis. |
| 2 | The median of Conv_T equals .00. | One-Sample Wilcoxon Signed Rank Test | .011 | Reject the null hypothesis. |
| 3 | The median of Acc_T equals .00. | One-Sample Wilcoxon Signed Rank Test | .109 | Retain the null hypothesis. |
| 4 | The median of Prac_T equals .00. | One-Sample Wilcoxon Signed Rank Test | .011 | Reject the null hypothesis. |
| 5 | The median of Asb_T equals .00. | One-Sample Wilcoxon Signed Rank Test | .012 | Reject the null hypothesis. |
| 6 | The median of App_T equals .00. | One-Sample Wilcoxon Signed Rank Test | .131 | Retain the null hypothesis. |
| 7 | The median of Eff_T equals .00. | One-Sample Wilcoxon Signed Rank Test | .180 | Retain the null hypothesis. |
| 8 | The median of Efc_T equals .00. | One-Sample Wilcoxon Signed Rank Test | .014 | Reject the null hypothesis. |
| 9 | The median of Saf_T equals .00. | One-Sample Wilcoxon Signed Rank Test | .440 | Retain the null hypothesis. |
| 10 | The median of Fit_T equals .00. | One-Sample Wilcoxon Signed Rank Test | .109 | Retain the null hypothesis. |
| a. The significance level is .050. | | | | |
| b. Asymptotic significance is displayed. | | | | |

| **Table S2. Client Unified Theory of Acceptance and Use of Technology one-sample Wilcoxon signed-rank test summary comparing median score and a reference score of 12 (total score of three questions asked based on Likert scale 1-7).** | | | | |
| --- | --- | --- | --- | --- |
|  | Null Hypothesis | Test | Sig.^a,b^ | Decision |
| 1 | The median of utautPE_taps equals 12.00. | One-Sample Wilcoxon Signed Rank Test | .011 | Reject the null hypothesis. |
| 2 | The median of utautEE_taps equals 12.00. | One-Sample Wilcoxon Signed Rank Test | .032 | Reject the null hypothesis. |
| 3 | The median of utautSI_taps equals 12.00. | One-Sample Wilcoxon Signed Rank Test | .670 | Retain the null hypothesis. |
| 4 | The median of utautFC_taps equals 12.00. | One-Sample Wilcoxon Signed Rank Test | .368 | Retain the null hypothesis. |
| 5 | The median of utautBI_taps equals 12.00. | One-Sample Wilcoxon Signed Rank Test | .057 | Retain the null hypothesis. |
| 6 | The median of utautUB_taps equals 12.00. | One-Sample Wilcoxon Signed Rank Test | .182 | Retain the null hypothesis. |
| a. The significance level is .050. | | | | |
| b. Asymptotic significance is displayed. | | | | |

**Table S3.** Client outcome measure score significance following Benjamini-Hochberg procedure to control False Discovery Rate and correct for multiple comparisons.

| **Variable** | **K** | **P-value**  **(*significant after correction)** | **Benjamini-Hochberg p-value Correction** |
| --- | --- | --- | --- |
| Client AQMH Convenience | 1 | 0.0110* | 0.0031 |
| Client AQMH Practicality | 2 | 0.0110* | 0.0063 |
| Client UTAUT Performance Expectancy | 3 | 0.0110* | 0.0094 |
| Client AQMH Accessibility | 4 | 0.0120* | 0.0125 |
| Client AQMH Efficiency | 5 | 0.0140* | 0.0156 |
| Client UTAUT Effort Expectancy | 6 | 0.0320 | 0.0188 |
| Client UTAUT Use Behavior | 7 | 0.0570 | 0.0219 |
| Client AQMH Fit | 8 | 0.1090 | 0.0250 |
| Client AQMH Acceptability | 9 | 0.1090 | 0.0281 |
| Client AQMH Appropriateness | 10 | 0.1310 | 0.0313 |
| Client AQMH Effectiveness | 11 | 0.1800 | 0.0344 |
| Client UTAUT Use Behavior | 12 | 0.1820 | 0.0375 |
| Client AQMH Ease of Use | 13 | 0.2010 | 0.0406 |
| Client UTAUT Facilitating Conditions | 14 | 0.3680 | 0.0438 |
| Client AQMH Safety | 15 | 0.4400 | 0.0469 |
| Client UTAUT Social Influence | 16 | 0.6700 | 0.0500 |
